# Supplementary material for: Heart Failure Promotes Cancer Progression in an Integrin β1-Dependent Manner
Source: Int J Mol Sci. 2023 Dec 11;24(24):17367. doi: 10.3390/ijms242417367 (PMC10744241; doi:10.3390/ijms242417367)
Supplement: Supplementary file 1 [file ijms-24-17367-s001.zip › ijms-2749610-supplementary.pdf]

Supplementary Figures and Legends

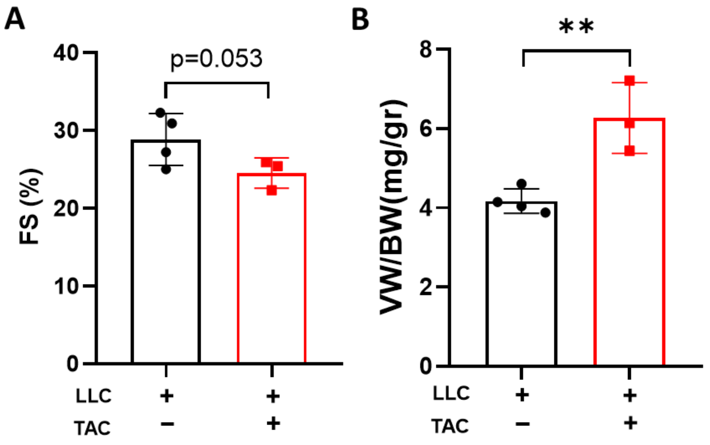

Supplementary Figure S1. POSTN$^{(-/-)}$ male mice cardiac remodeling following TAC (TAC-operated group; red, *n* = 3, and non-operated group: black, *n* = 4). (**A**). Echocardiography was performed one day before the sacrifice. Fractional shortening (FS) was assessed according to: FS (%) = [(LVDd-LVDs)/LVDd].
(**B**). Ventricular weight (VW) to body weight (BW) ratio. Data are presented as mean±SE. Student t-test (**A**,**B**). **P<0.01.

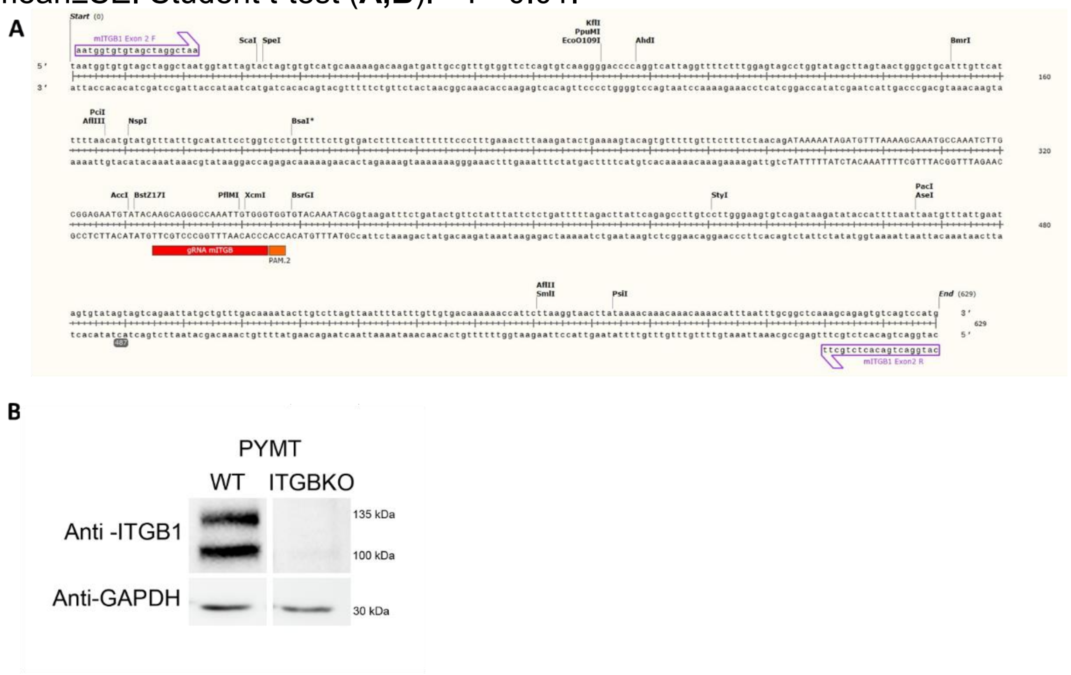

Supplementary Figure S2. CRISPR Cas-9 strategy for PyMT Integrin β1 KO breast cancer cell line. (**A**) Murine *Integrin β1* gene map of exon 2, showing the design of the gRNA used as the CRISPR target (red), and forward and reverse primers used to amplify the targeted region for Sanger sequencing (purple). (**B**) Western blot analysis of cell lysate derived from PyMT WT and PyMT ITGB1 KO clone. The blot was probed with anti-ITGB1 (upper) and anti-GAPDH was used as a loading control.

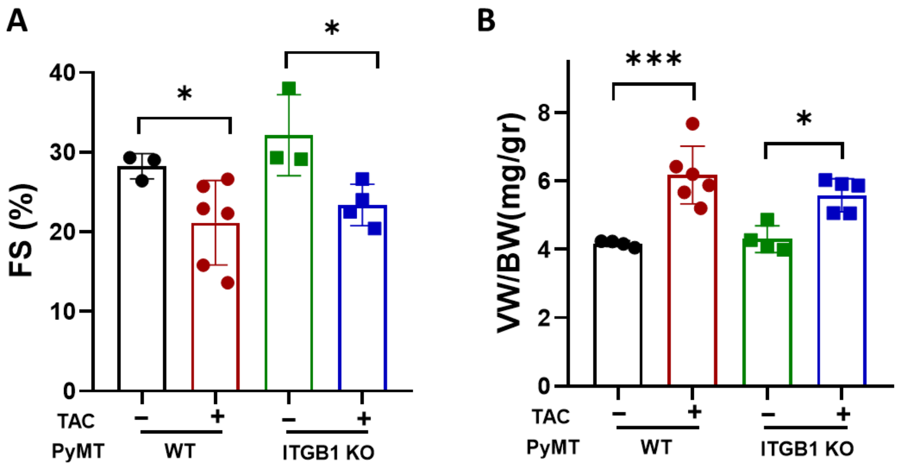

Supplementary Figure S3. Cardiac remodeling of C57Bl/6 female mice following TAC (WT PyMT; black, *n* =3, WT PyMT+TAC; red, *n* = 5, PyMT ITGB1 KO; green, *n* = 3, PyMT ITGB1 KO+TAC; blue, *n* = 5). (**A**) Echocardiography was performed one day before the sacrifice. Fractional shortening (FS) was assessed according to FS (%) = [(LVDd-LVDs)/LVDd]. (**B**) Ventricular weight (VW) to body weight (BW) ratio. Each dot represents one mouse. Data are presented as mean±SE. One-way ANOVA followed by

Bonferroni posttests (**A-B**) *P<0.05, ***P<0.001.

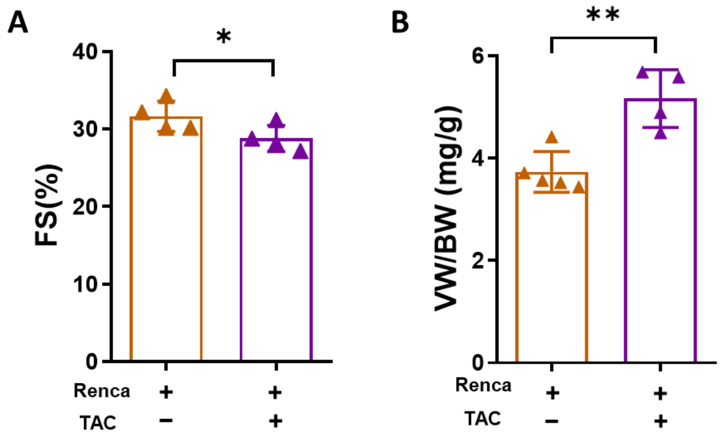

Supplementary Figure S4. Cardiac remodeling of C57Bl/6 female mice following TAC. TAC-operated group (purple, *n* = 4), and non-operated group (orange, *n* = 4). (**A**) Echocardiography was performed one day before the sacrifice. Fractional shortening (FS) was assessed according to: FS (%) = [(LVDd-LVDs)/LVDd]. (**B**) Ventricular weight (VW) to body weight (BW) ratio. Each dot represents one mouse. Data are presented as mean±SE. Student t-test (A,B). *P<0.05. **P<0.01.
